# *Bdellovibrio bacteriovorus* directly attacks *Pseudomonas aeruginosa* and *Staphylococcus aureus* Cystic fibrosis isolates

**DOI:** 10.3389/fmicb.2014.00280

**Published:** 2014-06-05

**Authors:** Valerio Iebba, Valentina Totino, Floriana Santangelo, Antonella Gagliardi, Luana Ciotoli, Alessandra Virga, Cecilia Ambrosi, Monica Pompili, Riccardo V. De Biase, Laura Selan, Marco Artini, Fabrizio Pantanella, Francesco Mura, Claudio Passariello, Mauro Nicoletti, Lucia Nencioni, Maria Trancassini, Serena Quattrucci, Serena Schippa

**Affiliations:** ^1^Microbiology Section, Department of Public Health and Infectious Diseases, “Sapienza” UniversityRome, Italy; ^2^Department of Pediatrics and Neuropsychiatry, “Sapienza” UniversityRome, Italy; ^3^Sapienza Nanoscience and Nanotecnology Laboratories, Department of Fundamental and Applied Sciences for Engineering, “Sapienza” UniversityRome, Italy; ^4^Section of Microbiology, Department of Biomedical Sciences, University G. D'AnnunzioChieti, Italy

**Keywords:** *Bdellovibrio bacteriovorus*, *Staphylococcus aureus*, *Pseudomonas aeruginosa*, biofilm, predation, Cystic fibrosis, FESEM

## Abstract

*Bdellovibrio bacteriovorus* is a predator bacterial species found in the environment and within the human gut, able to attack Gram-negative prey. Cystic fibrosis (CF) is a genetic disease which usually presents lung colonization by *Pseudomonas aeruginosa* or *Staphylococcus aureus* biofilms. Here, we investigated the predatory behavior of *B. bacteriovorus* against these two pathogenic species with: (1) broth culture; (2) “static” biofilms; (3) field emission scanning electron microscope (FESEM); (4) “flow” biofilms; (5) zymographic technique. We had the first evidence of *B. bacteriovorus* survival with a Gram-positive prey, revealing a direct cell-to-cell contact with *S. aureus* and a new “epibiotic” foraging strategy imaged with FESEM. Mean attaching time of HD100 to *S. aureus* cells was 185 s, while “static” and “flow” *S. aureus* biofilms were reduced by 74 (at 24 h) and 46% (at 20 h), respectively. Furthermore, zymograms showed a differential bacteriolytic activity exerted by the *B. bacteriovorus* lysates on *P. aeruginosa* and *S. aureus*. The dual foraging system against Gram-negative (periplasmic) and Gram-positive (epibiotic) prey could suggest the use of *B. bacteriovorus* as a “living antibiotic” in CF, even if further studies are required to simulate its *in vivo* predatory behavior.

## Introduction

Cystic Fibrosis is a lethal genetic disease (Davis et al., 1996; Lyczak et al., 2002) in which mutations in the CF transmembrane Conductance Regulator (*CFTR*) gene result in defective function and/or processing of the mutant protein CFTR (Zielenski and Tsui, 1995; Gadsby et al., 2006). Patients are prone to chronic, persistent, and recurrent respiratory tract infections, with an exaggerated inflammatory response, leading to progressive respiratory deficiency (Boucher, 2002; Dakin et al., 2002; Rajan and Saiman, 2002). Even if the lung is usually inhabited by various bacterial species (Goddard et al., 2012), in CF disease only one or two pathogenic species prevail (Moore et al., 2005; Harrison, 2007; Sibley and Surette, 2011), usually the Gram-negative *P. aeruginosa* and the Gram-positive *S. aureus*. These two species are able to establish chronic infections through biofilm formation and resistance, leading to clinical exacerbations (Lyczak et al., 2002; Rajan and Saiman, 2002). Due to the polymicrobial nature of healthy lung microbiota and, conversely, to the predominance of only one/two pathogenic species in CF (Moore et al., 2005), it was suggested how manipulating lung communities would be effective against chronic infections (Harrison, 2007). From literature it was reported that a bacterial predator, *Bdellovibrio bacteriovorus*, is capable to attack different Gram-negative bacterial genera (*Escherichia, Salmonella, Legionella, Pseudomonas*) and their pre-formed biofilms (Sockett and Lambert, 2004; Kadouri and O'Toole, 2005). *B. bacteriovorus* is ubiquitous in the environment, where it's alleged to act as an “ecological balancer species” (Varon, 1981; Yair et al., 2003; Dwidar et al., 2012), and it was recently found in the human gut of all healthy individuals examined (Iebba et al., 2013). *B. bacteriovorus* has the natural ability to predate Gram-negative bacteria by invading their periplasmic space, where it undergoes a complex replication cycle culminating in prey killing and release of progeny (Rendulic et al., 2004; Lambert et al., 2006). Lytic action exerted by *B. bacteriovorus* can rapidly reduce prey populations, making this predatory species, or its lytic enzymes (Dori-Bachash et al., 2008; Lambert et al., 2010; Lerner et al., 2012), potential therapeutic candidates (Sockett and Lambert, 2004; Dwidar et al., 2012). Research is still necessary to give insights into the predatory spectrum of *Bdellovibrio*. The present study aimed at evaluating the predatory activity of *B. bacteriovorus* strain HD100 on bacterial strains commonly isolated from CF patients, such as the Gram-negative *P. aeruginosa* and the Gram-positive *S. aureus*.

## Materials and methods

### Bacterial strains and cultivation

The predator *Bdellovibrio bacteriovorus* strain HD100 (DSM No. 50701), was acquired by the German microorganisms collection DSMZ (Braunschweig, Germany), and arrived in our lab in a “double-layer agar plate” containing diluted Nutrient-broth (NB) (1:10 dilution of NB amended with 3 mM MgCl_2_•6H_2_O and 2 mM CaCl_2_•2H_2_O [pH 7.2]) and agar (0.6% upper layer) with enclosed prey cells of *P. aeruginosa* and *B. bacteriovorus* forming lysis plaque (Starr, 1975). Prey bacterial strains (*P. aeruginosa* and *S. aureus*) used in this study were recovered from glycerol stocks stored at −80°C, and were previously collected from sputa of two Cystic fibrosis patients with chronic mono-colonization attending the Cystic fibrosis center of Lazio Region, “Sapienza” University of Rome. All strains were identified with the automated Vitek 2 system (Biomèrieux, Marcy l'Etoile, France). Strains were first suspended in Tryptone Soya Broth (TSB), and, to verify the purity of the culture, 100 μL of overnight culture were spread onto Tryptone Soya Agar (TSA) plates. Vitek2 assessed strain identification again. The predator strain *B. bacteriovorus* was grown as previously reported (Kadouri and O'Toole, 2005; Jurkevitch, 2006; Lambert and Sockett, 2008) in DNB minimal medium. Briefly, DNB growth medium contained: 0.8 g/L Bacto Nutrient Broth (NB) and 0.1 g/L yeast extract, with the separated addition of 0.45 μm-filtered 0.3 g/L of Casaminoacids, 0.5 g/L of CaCl_2_ × 2H_2_O and 0.6 g/L of MgCl_2_ × 6H_2_O. To obtain an enriched predator preparation to be used in predation assays, we modified the cultivation media doubling the concentration of NB from 0.8 to 1.6 g/L, and the resulting medium was hereafter named “2X DNB.”

### Preparation of *B. bacteriovorus* suspension for predatory assays

Two small pieces of agar were removed from a commercially available “double-layer agar plate” of *B. bacteriovorus* preying on *P. aeruginosa*, as recommended by DSMZ instructions, and added to 60 mL of “2X DNB.” Incubation was performed at 30°C under agitation (180 rpm) for 48 h. Through microscopic observations at 100X magnification and the hanging drop technique, we were able to follow and measure every 2 h the growth of *B. bacteriovorus*, discernible by a reduction in OD_600_ turbidity (clear lysate). Upon reaching 48 h, 30 mL of the clear lysate were 0.45 μm-filtered for three times in order to remove prey cells. One-hundred μL of the filtrate were plated on TSA agar plates, and incubated at 37°C overnight to exclude carryover of the prey (*P. aeruginosa*). Finally, to have a three-times concentrated suspension of *B. bacteriovorus* and to remove the “2X DNB” broth, which initial tests showed to interfere with prey biofilm (data not shown), the 0.45 μm filtrate was centrifuged at 10,000 g for 30 min and pellet was suspended in 10 mL of TSB. *B. bacteriovorus* suspension was prepared fresh each time for subsequent experiments.

### Predation assays on prey cultures

One colony of prey (*P. aeruginosa* or *S. aureus*) was picked up from TSA plates and grown overnight in 20 mL of TSB at 37°C, 200 rpm. Bacterial culture was centrifuged (5000 g, 15 min) and pellet suspended in pre-warmed TSB till reaching an OD_600_ = 1. One-hundred μL of suspended culture were used to inoculate 60 mL of pre-warmed TSB, and incubated at 30°C with shaking (200 rpm). Bacterial growth was followed each hour with a spectrophotometer (BioPhotometer, Eppendorf, Hamburg, Germany), and at OD_600_ = 1 the prey culture was split into three different 100 mL-flasks at equal volumes (20 mL): (1) the first left as it is; (2) the second added with 2 mL of *B. bacteriovorus* suspension (see “*B. bacteriovorus* suspension for predatory assays” paragraph); (3) the third added with 2 mL of *B. bacteriovorus* suspension 0.22 μm-filtered (for assessing the action of lytic enzymes eventually released into the medium). Flasks were incubated at 30°C with shaking (200 rpm) into the same incubator/shaker GFL 3031 (MicroGlass Heim, Naples, Italy) and bacterial growth was measured every hour in two different ways: (1) OD_600_ (BioPhotometer, Eppendorf, Hamburg, Germany); (2) bright field through the “hanging drop” technique (optical microscope DM 5000-D, Leica Microsystems, Wetzlar, Germany). Prey levels (*P. aeruginosa* and *S. aureus*) were assessed plating every 2 h, on TSA plates, 100 μL taken from the abovementioned first and second flasks, along with appropriate serial dilutions: colonies formed on plates after 17 h at 37°C were automatically counted by means of the TotalLab TL120 software (Non-linear Dynamics), “colony counting” module. Predation assays in TSB broth were performed in triplicate: OD_600_ values and colony forming units (CFU) per mL were expressed as mean ± SD of the mean. Graphs and statistical tests were done with Prism 5 software (GraphPad, La Jolla, California, USA).

### Predatory activity of *B. bacteriovorus* on “static” biofilms

Biofilms of *P. aeruginosa* and *S. aureus* were pre-formed on 48-well plates as already reported (Merritt et al., 2005). Briefly, 200 μL of prey overnight cultures diluted in TSB at OD_600_ = 1 were used to inoculate the 48-well plate, followed by incubation at 37°C for 24 h. Planktonic bacteria were removed by Phosphate Buffered Saline (PBS) washing. After washing, 200 μL of *B. bacteriovorus* preparation (see “Preparation of *B. bacteriovorus* for predatory assays” paragraph) were added to ½ plate, while ¼ was added with 200 μL of 0.22 μm-filtered *B. bacteriovorus* preparation, and another ¼ was added with 200 μL of TSB (control). Plates were incubated at 37°C for additional 24 h to allow *B. bacteriovorus* predation against prey biofilm. Then, all wells were washed with PBS for three times, then 100 μL of 1% crystal violet were added to each well and left in contact for 5 min. The dye in excess was eliminated by three washes with PBS, and the plate dried in a thermostat. 250 μL of 33% glacial acetic acid were added to each well and left in contact for 15 min. Subsequent OD_570_ readings were done with VMax® Kinetic Microplate Reader (Molecular Devices, Sunnyvale, CA, USA). Wilcoxon Signed Rank test was employed to assess differences in biofilm amount, and a *P*-value less than or equal to 0.05 was considered statistically significant. Graphs and statistical tests were done with Prism 5 software (GraphPad, La Jolla, California, USA).

### Field-emission scanning electron microscopy (FESEM) of *S. aureus* biofilms

FESEM technique was employed to visualize *S. aureus* biofilm before and after a 24 h-challenge of *B. bacteriovorus*. As a substrate for FESEM microscopy, double-sided polished silicon wafers were reduced into many thin pieces by using a diamond cutter along natural crystallographic lines. Pieces of silicon wafers (hereafter mentioned as “wafers”) were sterilized at 121°C for 15 min and then aseptically used as a substrate for bacterial biofilm growth. Wafers were separately immersed in 5 mL of 10^6^ CFU/mL of *P. aeruginosa* or *S. aureus* broth cultures, respectively. After 24 h of incubation at 37°C, 180 rpm, the supernatant and the planktonic preys were gently removed and replaced by *B. bacteriovorus* suspension (see “*B. bacteriovorus* suspension for predatory assays” paragraph). The predator was left in presence of the different prey-biofilms for additional 24 h. After 24 h of contact between predator and preys, all wafers were gently rinsed by sterile saline solution (PBS) and immediately immersed in a fixative solution consisting of glutaraldehyde and PBS (2% v/v). At this step, all samples were maintained in the dark at 25°C for 1 h, then washed three times in PBS and immediately after immersed in osmium tetroxide 1% aqueous solution (cat# 75632-10 ML, Sigma-Aldrich, St. Louis, MO, USA) and kept in the dark for 24 h at 4°C. To obtain the necessary dehydration, after three PBS washes, wafers were immersed for 10 min each time in subsequent ethanol solutions increasing progressively in concentration from 30 to 99% (30–50–70–80–90–99%). After drying at room temperature, samples were observed by FESEM microscopy. Identical preparation procedure has been adopted for all the control samples without predator or prey. FESEM images were captured using a Zeiss Auriga 405 (Carl Zeiss AG, Oberkochen, Germany). Different extraction voltages (5–10 keV) and a specific working distance (around 4 mm) were employed to find an affordable compromise among avoiding the radiation damage and enhancing the contrast between bacterial cells and siliceous substrate. FESEM images were captured without any additional surface coating of the sample, in order to evaluate the actual surface morphology of bacterial cells.

### Predatory activity of *B. bacteriovorus* on “flow” biofilms

BioFlux system (Fluxion Biosciences, South San Francisco, CA) was used to visualize and follow in a time-dependent manner the development and the subsequent predatory activity of *B. bacteriovorus*. Microfluidic plates used were BioFlux 200 WPM 24 well plates 0–20 Dynes (cat. #910-0009), and instrumental setup was set following manufacturer's instructions and literature (Benoit et al., 2010). Two wells were inoculated with 800 μL of a suspension of the bacterial prey (*P. aeruginosa* or *S. aureus*) in TSB (OD_600_ = 1), to allow biofilm formation within each microfluidic channel. An initial pressure of 2 dyne/cm^2^ for 4 s was applied in the upper side of the inlet allowing the initial flow of bacterial inoculum. Flow was then stopped for 30 min to allow the initial stages of biofilm formation, then 2 mL of pre-warmed TSB medium were added, and flow started again at 0.5 dyne/cm^2^, 37°C for 24 h. Pre-formed biofilm was then added with 2 mL of *B. bacteriovorus* preparation (upper channel), while the control microfluidic well (lower channel) was added with 2 mL of TSB. When the *B. bacteriovorus* preparation and TSB were added, the flow was started again at 0.5 dyne/cm^2^, 37°C for 24 h. Still frames were taken every minute during the entire procedure (biofilm formation, predation) with a QICAM 12-bit camera (QImaging, Surrey, Canada) at a resolution of 1392 × 1040 pixels (4.65 × 4.65 μm pixel size) and 12-bit of image depth. By means of a specific software (ImageJ, National Institutes of Health, USA), measurements of gray intensity were made for each frame in a 0–255 scale, where 0 is black and 255 is white. Due to the different bacterial cell sizes of *P. aeruginosa* (rod-shaped, mean length and width, 3.0 and 0.8 μm, respectively), *S. aureus* (spherical, mean diameter 1.0 μm), and *B. bacteriovorus* (rod-shaped, mean length and width, 1.2 and 0.4 μm, respectively), the gray intensity value given by *B. bacteriovorus* alone was resulted to be 240.7 ± 1.8 (with a component of around 4% on the total mean gray intensity), and such a value was subtracted from each frame. Experiments were performed in triplicate by the same operator, while gray intensity measures were done in triplicate on each still frame from three different operators. Wilcoxon Signed Rank test was employed to assess differences in mean gray intensity on each movie frame, and a *P*-value less than or equal to 0.05 was considered statistically significant. Graphs and statistical tests were done with Prism 5 software (GraphPad, La Jolla, California, USA).

### Zymographic technique

The zymographic is an electrophoretic technique which includes a substrate copolymerized in a polyacrylamide gel useful for the detection of enzyme activity. Zymographic technique included the use of fresh lysates of *B. bacteriovorus* loaded on polyacrylamide gel copolymerized with prey cells, and was used as stated in literature (Audy et al., 1989; Lantz and Ciborowski, 1994) with minor modifications. Briefly, polyacrylamide mini-gels contained 10% polyacrylamide, Tris-HCl (pH 8.8), sodium-dodecyl-sulphate (SDS) 0.1%, and 15% of prey cell suspension at OD_600_ = 20. This huge bacterial density was necessary in order to hinder the action exerted by SDS, especially for *P. aeruginosa*. The *B. bacteriovorus* preparation was sonicated 10 times with a 300VT ultrasonic homogenizer (Biologics Inc., Manassas, Virginia, USA) to disrupt predator cells and bring enzymes in solution. Then, an equal volume of sample buffer (Bromophenol blue in 20% glycerol in 2:1 ratio) was added to the sonicated *B. bacteriovorus*, and loaded. The run was set with a constant voltage (120 volts for 1 h). Renaturation of proteins in polyacrylamide gel was obtained by washing the gel for 3 h, with changes of the solution every 30 min, in renaturing buffer (50 mM Na-phosphate buffer at pH 7 with 1% Triton X-100). After the 3 h of washings, the gel was left in renaturing buffer for additional 24 h at 37°C. Gel was stained with 0.25% (w/w) Coomassie Brilliant Blue R-250. Clear band on opalescent gel matrix indicated bacteriolytic activities. Zymograms were analyzed by TotalLab TL120 software (Non-linear Dynamics) and molecular weights of unknown lytic bands were inferred by a logarithmic interpolation of marker bands (unstained SDS-PAGE low range standard, Bio-Rad cat #161-0304).

## Results

### *B. bacteriovorus* HD100 predation on *P. aeruginosa* and *S. aureus* in broth culture

**Figure 1:**
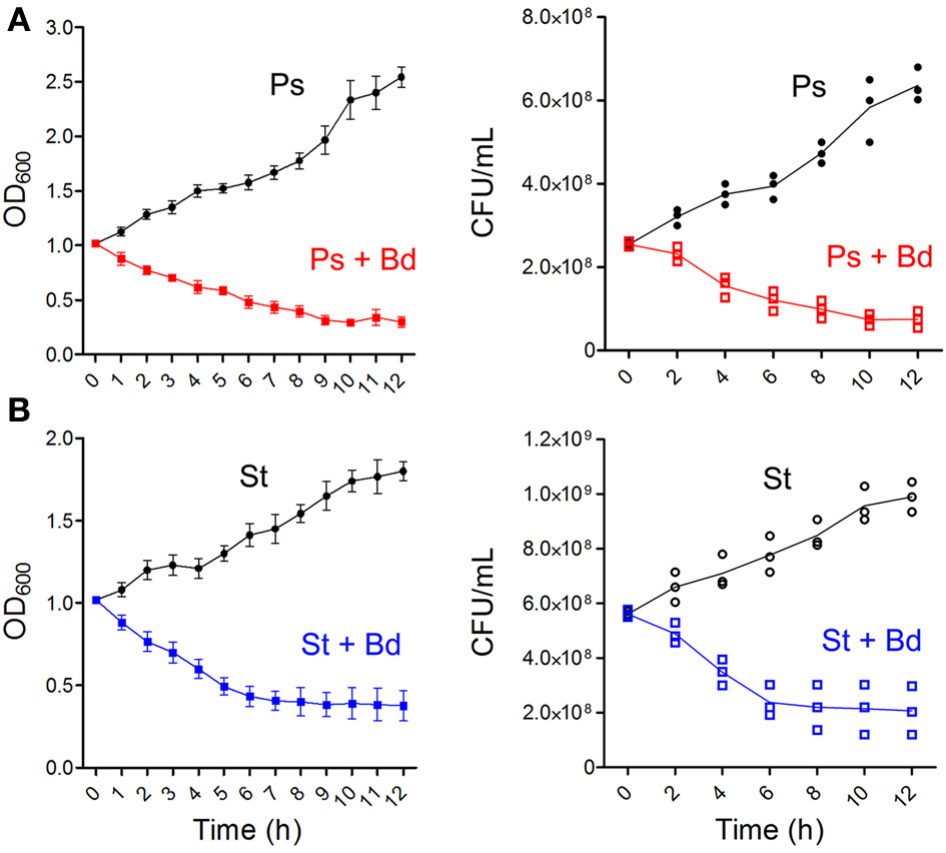
***B. bacteriovorus* predation on broth cultures of *P. aeruginosa* and *S. aureus***. Prey species [*P. aeruginosa*, red **(A)**; *S. aureus*, blue **(B)**] were grown in TSB till OD_600_ = 1, then 2 mL of *B. bacteriovorus* suspension were added to 20 mL of culture. Treated and control flasks were in the same incubator at 37°C and 200 rpm. OD_600_ measurements were done in triplicate every hour, while CFU/mL counts were done plating 100 μL of culture (along with serial dilutions) onto TSA plates, leaving colonies to grow overnight at 37°C.

**Figure 2:**
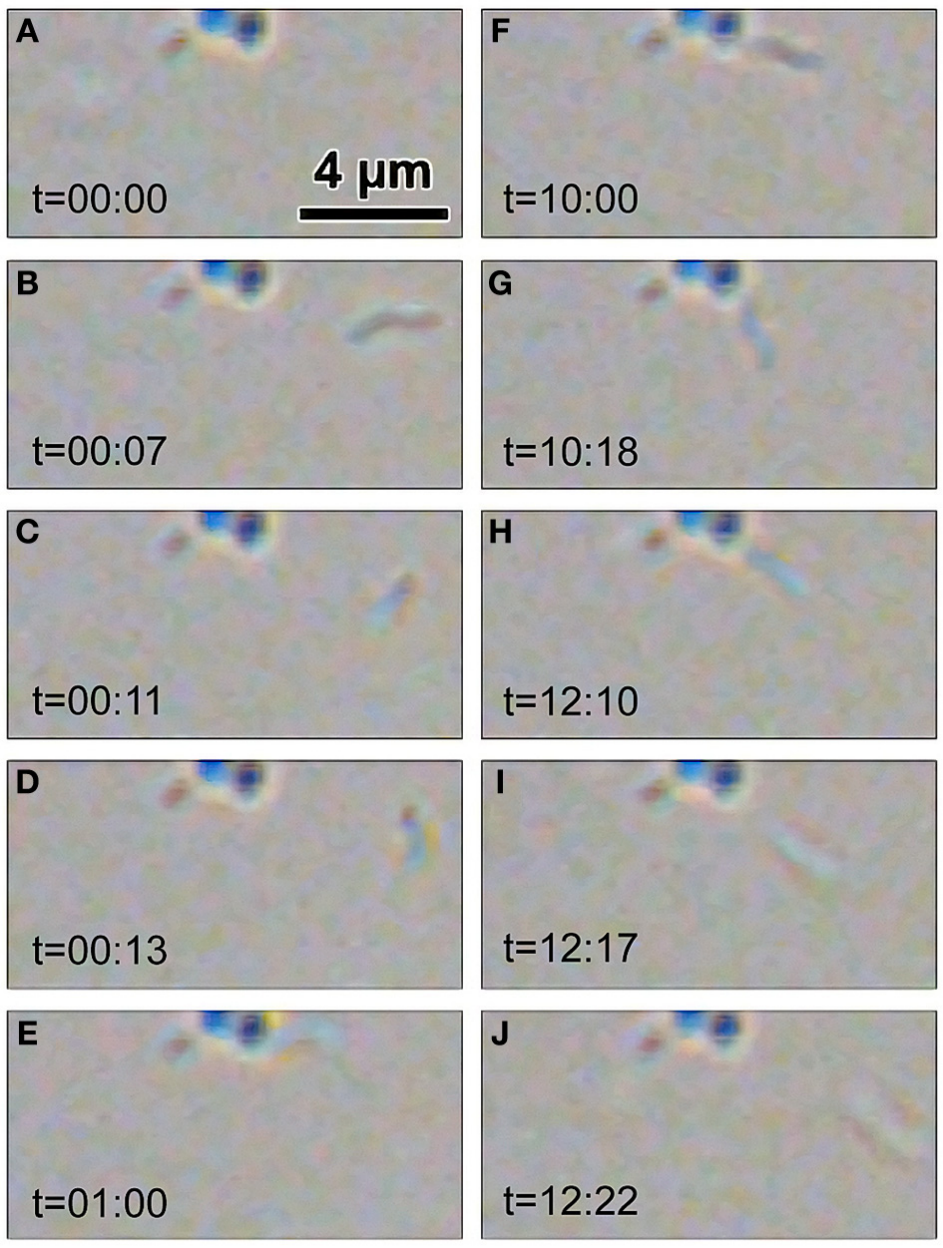
***B. bacteriovorus* predation on two *S. aureus* prey cells**. Frames **(A–J)** from Supplementary Video S1 (bright field, 100X magnification) depicting predation phases of *B. bacteriovorus* on two *S. aureus* cells. Time (t) is expressed as “seconds:frames,” where each second is made of 25 frames. A single *B. bacteriovorus* enters from the left, attacks its prey (right, dark blue) and after around 12 s moves away. A second *B. bacteriovorus* is already attached to its prey (left, bright blue), perpendicularly to the frame level during the entire Supplementary Video S1.

A first assessment of *B. bacteriovorus* predatory activity was done on prey species (*P. aeruginosa* and *S. aureus*) in TSB broth. Predation curve of *B. bacteriovorus* on *P. aeruginosa* reached stabilization at OD_600_ = 0.30 (around 7.5 × 10^7^ CFU/mL) after 10 h, and this turbidity level was stable till the end of assay (Figure 1A). Interestingly, we observed predation of *B. bacteriovorus* against the Gram-positive *S. aureus* (Figure 1B), and in this case the predation curve stabilized itself at OD_600_ = 0.38 (around 2.2 × 10^8^ CFU/mL) after 7 h. A slight reduction in OD_600_ measurements was visible when 0.22 μm-filtered suspension of *B. bacteriovorus* was added to prey cultures, but this reduction was not significant in both prey species (data not shown). Due to the unexpected observation of *B. bacteriovorus* HD100 predation on *S. aureus* in broth culture, we decided to see its actual predatory behavior utilizing the “hanging drop” technique and making a movie with an optical microscope (bright field) at 100X magnification (Supplementary Video S1). In Figure 2 are reported eight frames from Movie S1 depicting four distinct phases of *B. bacteriovorus* predation on *S. aureus*: sensing (Figures 2A–C), attacking (Figures 2D,E), breaching (Figures 2F–H), and detaching (Figures 2I,J). As shown in Supplemental Movie (Supplementary Video S1) and Figure 2, while *B. bacteriovorus* swims at high speed from a lower depth level (Figure 2A), it surpasses *S. aureus* cells (Figure 2B), swiftly turns back (Figures 2C,D), and hooks one of them (Figure 2E), exhibiting a bending of its cellular body (Figure 2E). After twisting for around 11 s trying to breach its prey (Figures 2F–H), *B. bacteriovorus* detaches from Gram-positive prey cell (Figure 2I) and moves away (Figure 2J). Time spent by *B. bacteriovorus* during the breaching phase on *S. aureus* (from attacking till detaching) was computed for different predator/prey couples, and resulted to be 185.5 ± 25.7 s (mean ± SD of the mean). Interestingly, predators who spent more time attached to *S. aureus* cells trying to breach them, in most cases were steady, with no detectable twisting (Figure 2 and Supplementary Video S1, bright blue prey; Supplementary Video S2). Such an observation shows an unconventional predatory behavior of *B. bacteriovorus* HD100, which usually holds on in twisting upon attaching Gram-negative prey to breach and penetrate into periplasmic space (Medina et al., 2008). No bdelloplast formation was observed throughout the entire period of observation (12 h).

### *B. bacteriovorus* HD100 reduces “static” prey biofilms

**Figure 3:**
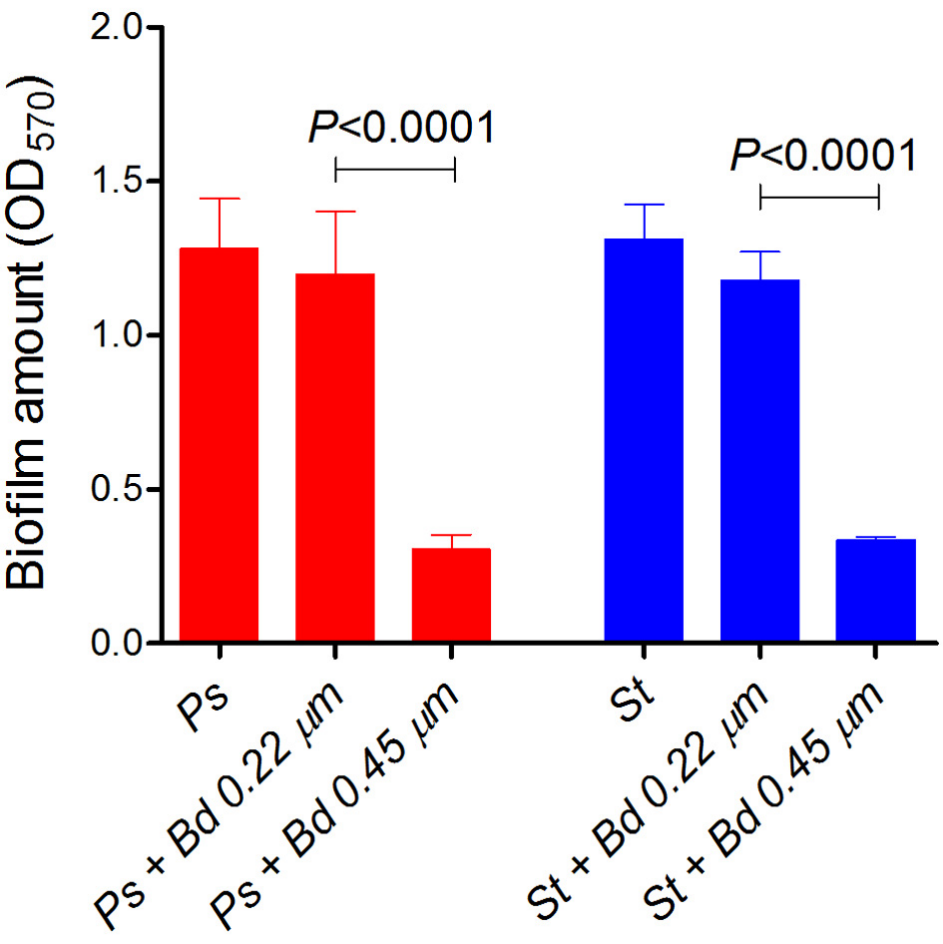
**Predation of *B. bacteriovorus* on “static” prey biofilm**. Biofilm of *P. aeruginosa* (Ps, red) and *S. aureus* (St, blue) were grown on 48-well plates for 24 h, and challenged with *B. bacteriovorus* for additional 24 h. After crystal-violet staining, OD_570*nm*_ was used to measure biofilm amount, and Wilcoxon Signed Rank test was used to assess statistical differences.

Twenty-four hours-old biofilms of *P. aeruginosa* and *S. aureus* strains were challenged for 24 h with *B. bacteriovorus* predation, and quantified by crystal violet staining followed by OD_570_ readings. *P. aeruginosa* biofilm was significantly reduced of 76%, while *S. aureus* biofilm was reduced of 74% (*P* < 0.0001, Figure 3). A prey preference of *B. bacteriovorus* for *P. aeruginosa* was visible, whose biofilm was reduced 9.3% more than *S. aureus* (*P* = 0.0437) (Figure 3). Even if significantly different, the similar biofilm amount of the two challenged species could indicate a similar predatory level exerted by *B. bacteriovorus*, irrespective from presence or absence of periplasmic replication. Biofilm amount in both prey species was not significantly affected by 0.22 μm-filtered suspension of *B. bacteriovorus* HD100, in which lytic enzymes would be eventually released (*P. aeruginosa, P* = 0.8667; *S. aureus, P* = 0.8596), signifying that a specific predator-prey interaction is needed to challenge pre-formed biofilms.

### *B. bacteriovorus* HD100 shows an epibiotic predation on *S. aureus*

**Figure 4:**
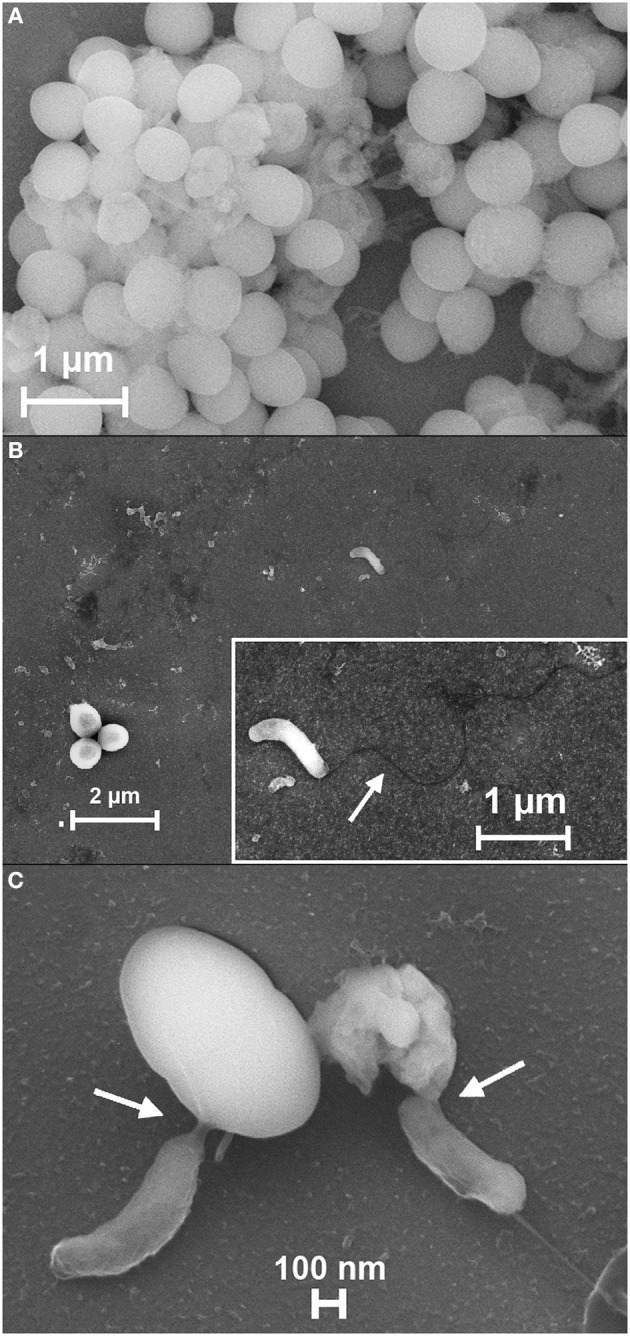
**Predation of *B. bacteriovorus* on “static” biofilm of *S. aureus***. s.e.m. images of *S. aureus* biofilm (**A**, 30000X) grown for 24 h on a silicon plate, and after 24 h of *B. bacteriovorus* HD100 predation (**B**, 20000X). A free *Bdellovibrio* is visible with its long polar flagellum (estimated length, 4 μm) (arrow, **B**, inset, 50000X). A higher s.e.m. magnification (88830X) shows the initial attack phase of HD100 on a prey cell (**C**, left) and the late attack phase, with a destroyed *S. aureus* cell (**C**, right). White arrows in **(C)** show the direct interaction of *B. bacteriovorus* HD100 with *S. aureus*.

Results obtained with “hanging drop” technique (Figure 2, Supplementary Video S1, Supplementary Video S2) allowed us to see the predatory behavior of HD100 toward *S. aureus*, thus, the subsequent step was to find out a better visualization of *B. bacteriovorus* attacking its Gram-positive prey. To this end, we employed FESEM technique on pre-formed biofilm of *S. aureus* before and after a challenge with *B. bacteriovorus*. Biofilm of *S. aureus* was grown on a siliceous slice for 24 h in TSB, then the medium was carefully removed and replaced with 5 mL of HD100 suspension (see “Preparation of *B. bacteriovorus* suspension for predatory assays”) allowing biofilm challenge for another 24 h. In Figure 4 are shown the results. A 24 h-old *S. aureus* biofilm was established (Figure 4A), but after 24 h the biofilm was thoroughly removed, leaving few prey cells alive surrounded by many debris (Figure 4B) with a single *B. bacteriovorus* approaching through the long (4 μm) flagellum (Figure 4B, inset). A higher FESEM magnification showed two *B. bacteriovorus* predators attacking two different *S. aureus* cells in an epibiotic manner, in which a direct contact (Figure 4C, white arrows) with prey cell is maintained throughout the entire predation process, from the initial attack phase (Figure 4C, left) till the late attack phase (Figure 4C, right). Interestingly, *B. bacteriovorus* HD100 was able to attack its prey with the anterior part, leaving the flagellum free to move, maybe aiding in pushing the predator body (Figures 2, 4C). This new behavior (epibiotic predation) against *S. aureus* is significantly different from what usually *B. bacteriovorus* HD100 does against Gram-negative prey (periplasmic predation).

### *B. bacteriovorus* HD100 reduces “flow” prey biofilms

**Figure 5:**
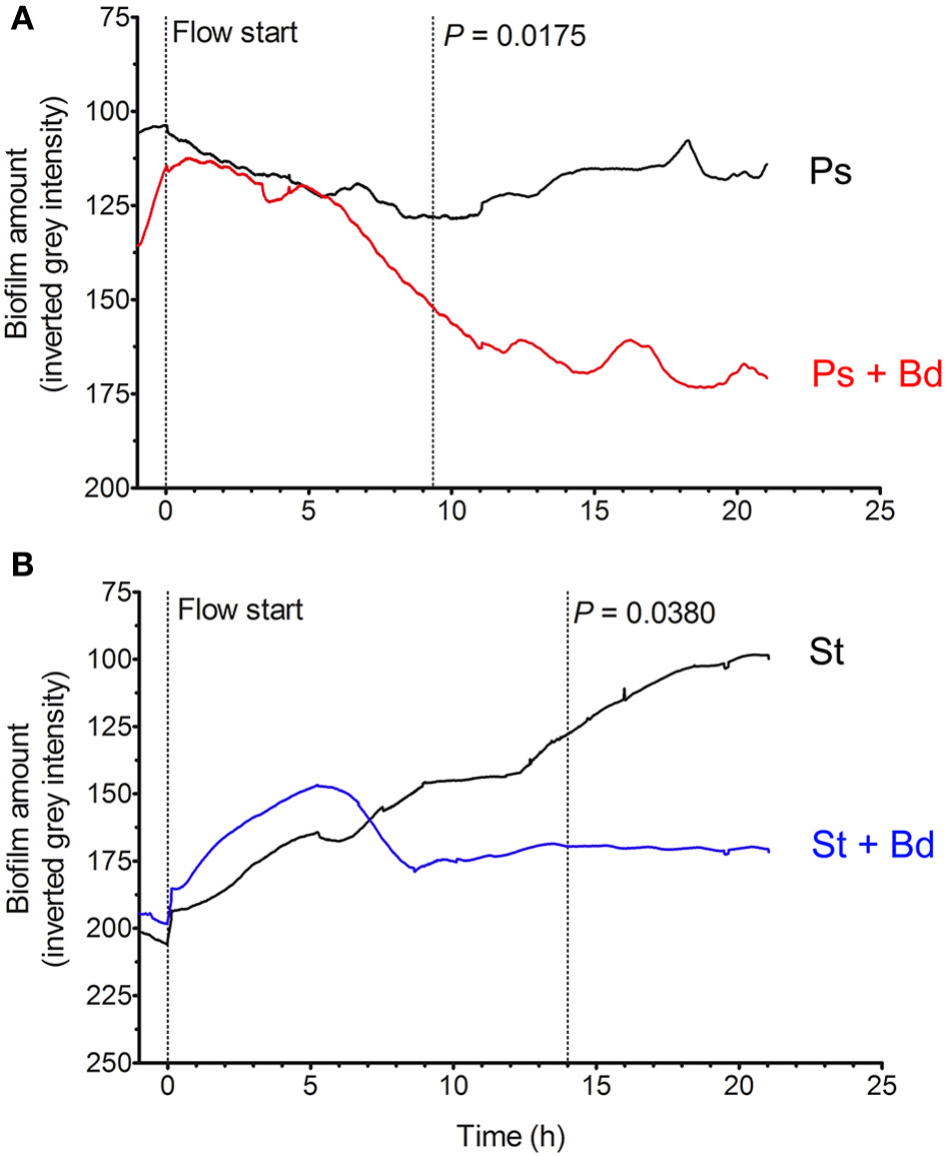
**Predation of *B. bacteriovorus* on “flow” prey biofilm**. Biofilms of *P. aeruginosa* **(A**, red**)** and *S. aureus* **(B**, blue**)** were grown in 24-well microfluidic plates (BioFlux), then challenged with *B. bacteriovorus* suspension for 24 h. Biofilm amount was expressed as mean gray intensity (from 255-white to 0-black) of each movie frame, and statistical differences were assessed by Wilcoxon Signed Rank test.

CF patients' lungs show a somewhat impaired mucociliary clearance (MCC), even if precise *in vivo* measurements lead to controversial results depending on upper/lower lobes involvement (Tarran et al., 2005): such a diminished MCC allows bacterial establishment and permanence in biofilms (Mall et al., 2004; Donaldson et al., 2007). We then hypothesized that a physiologic shear-stress of 0.5 dyne/cm^2^, as found in periciliary liquid (PCL) of healthy and CF individuals (Regnis et al., 1994; McShane et al., 2004; Tarran et al., 2005), could affect *B. bacteriovorus* predation of prey biofilms. To test such hypothesis, evaluation of *B. bacteriovorus* predatory activity on preformed biofilms was done in dynamic settings, by means of BioFlux microfluidics apparatus. The first significant reduction of *P. aeruginosa* biofilm (−31%) was visible after 9 h upon *B. bacteriovorus* challenge (*P* = 0.0175), while after 20 h it was reduced by 38% (Figure 5A). Interestingly, after a fixed decreasing rate (from 6 to 11 h) of *P. aeruginosa* biofilm amount (−6.3%/h), an oscillation with a period T_*Ps*_ = 4 h was visible starting around at 12 h, reminiscent the predator/prey Lotka-Volterra model (Varon and Zeigler, 1978). Such an oscillation was not due to the BioFlux peristaltic pump, which showed instead a period T_*pump*_ = 0.7 h. A significant reduction of *S. aureus* biofilm (−33%) was visible after 14 h of contact with the predator (*P* = 0.0380), and after 20 h it was reduced by 46% (Figure 5B). From 6 to 8.5 h a fixed decreasing rate of *S. aureus* biofilm amount (−17.5%/h) was observed, but no oscillations were visible thereafter, maybe owing to the absence of a host-dependent replicative cycle of *B. bacteriovorus*. Strikingly, as observed in static conditions, after 20 h of predation in flow settings both biofilm amounts reached a similar level of gray density (169.1 ± 18.0 for *P. aeruginosa*, 170.2 ± 7.4 for *S. aureus*, Figure 5), with no significant difference (*P* = 0.9269).

### *B. bacteriovorus* HD100 uses different non-released degradative enzymes to lyse *P. aeruginosa* and *S. aureus*

**Figure 6:**
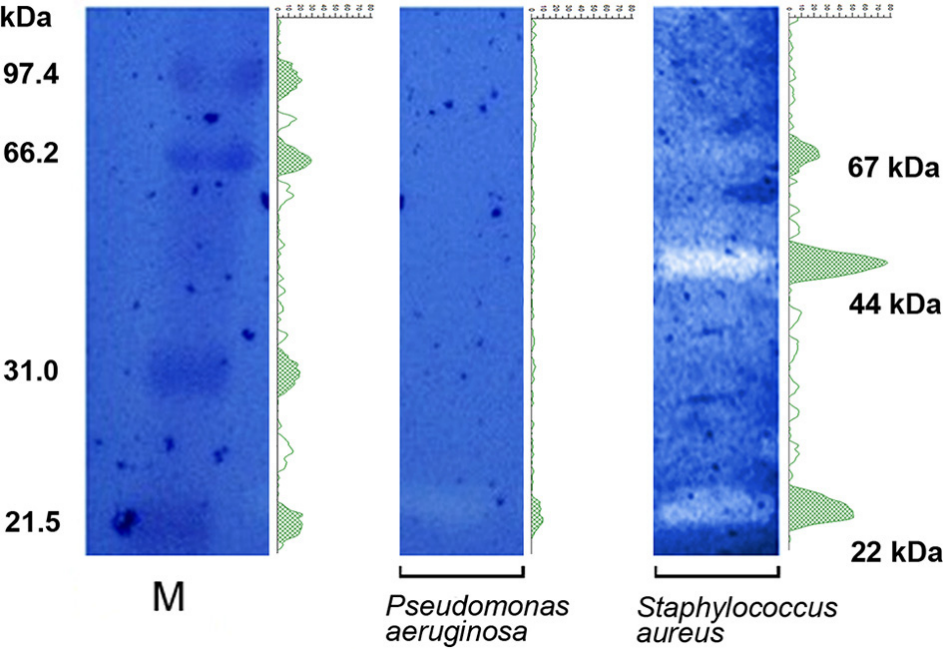
**Zymograms**. *P. aeruginosa* e *S. aureus* were embedded into different polyacrylamide gels and wells were loaded with sonicated *B. bacteriovorus*. Species-specific lytic bands (white) were visible in correspondence of *B. bacteriovorus*-derived lytic enzymes, after blue coomassie staining. Densitometry profiles (green) were added to appreciate the differential bacteriolytic activity exerted by *B. bacteriovorus* on prey cells. M, marker (unstained SDS-PAGE low range standard, Bio-Rad cat #161-0304).

Results obtained with the “hanging drop” technique (Figure 2, Supplementary Video S1, Supplementary Video S2), “static” biofilms (Figure 3), and FESEM images (Figure 4), suggested that a direct interaction of *B. bacteriovorus* HD100 is needed to ensure predation, and that its degradative enzymes eventually released into the medium do not exert a role in lysing neither free prey nor their pre-formed biofilms. Zymographic technique was used to find non-released bacteriolytic enzymes of *B. bacteriovorus* against cellular substrates of *S. aureus* or *P. aeruginosa*. Zymograms densitometry profiles were divided in 312 pixels and molecular weights of lytic bands (expressed as kDa) were inferred by a logarithmic interpolation of the marker (Log curve, *y* = 111.6*e*^−0.009371*x*^ + 14.66, *R*^2^ = 1). A differential bacteriolytic activity against *P. aeruginosa* and *S. aureus* was observed (Figure 6), with a unique clear band at around 22 kDa for the Gram-negative prey, and three different lytic bands at 22, 44, and 67 kDa for the Gram-positive prey (Figure 6). SDS added to polyacrylamide gels allowed us to be quite confident in assessing the molecular weights of lytic bands (Lantz and Ciborowski, 1994), especially for *S. aureus*, even if a proper characterization of enzymes involved will be done in forthcoming experiments.

## Discussion

Since its discovery in 1963 (Stolp and Starr, 1963), *Bdellovibrio* was recognized as a predator species capable to prey Gram-negative bacteria, maybe exerting a role in regulating microbial communities (Varon, 1981; Yair et al., 2003; Dwidar et al., 2012). Previous reports suggested to use *B. bacteriovorus*, or similar predatory species, as a biological agent against bacterial pathogens, and found it to be actually useful in challenging such infections (Chu and Zhu, 2010; Atterbury et al., 2011; Dashiff and Kadouri, 2011; Dashiff et al., 2011). CF is usually accompanied to an exaggerated bacterial colonization of the lower respiratory tract (Moore et al., 2005; Harrison, 2007; Sibley and Surette, 2011), in which *P. aeruginosa* and *S. aureus* trigger an inflammatory response that leads to progressive clinical exacerbation (Lyczak et al., 2002; Rajan and Saiman, 2002). The objective of the present study was to evaluate the predatory activity of *Bdellovibrio bacteriovorus* strain HD100 against two bacterial pathogens usually isolated from CF patients' sputa, *P. aeruginosa* and *S. aureus*. To this end, the activity of *B. bacteriovorus* was evaluated vs. cells prey in culture and on preformed biofilms, both in “static” and “flow” conditions. Bioflux results on *P. aeruginosa* revealed the instauration of a Lotka-Volterra predator/prey oscillation after 12 h of *B. bacteriovorus* attack: further experiments with fluorescent dyes will be addressed to ascertain the predator/prey ratio at the origin of this oscillation. Interestingly, the reduction rate of both Gram-negative and Gram-positive biofilms started at 6 h upon *B. bacteriovorus* challenge, and the final biofilm amount settled at around −38% in both prey species (Figure 5). We cannot exclude that *S. aureus* biofilm was still growing while *P. aeruginosa* biofilm was at its plateau (Figure 5), but we found that such a biofilm development and subsequent predation was reproducible under simulated physiologic shear-stress (“flow” condition) of 0.5 dyne/cm^2^ for both preys. “Flow” conditions diminished predatory activity of *B. bacteriovorus* by 38 (*P. aeruginosa*) and 28% (*S. aureus*) compared to “static” conditions, and this observation could be useful if an *in vivo* treatment would be employed for *B. bacteriovorus* in CF disease. In this view, further studies are needed to validate the effect of shear-stress in PCL on *B. bacteriovorus* predation by using CF animal models.

We found that *B. bacteriovorus* strain HD100 could survive for a prolonged time (at least 20 h) exclusively preying a Gram-positive species, here a *S. aureus* CF isolate. Previous papers reported how some bacterial predators genera, namely *Cupriavidus* (Casida, 1988), *Lysobacter* (Bonner et al., 1988), and *Myxococcus* (Shimkets, 1990), could prey Gram-positive preys in an epibiotic manner, but such an evidence was never reported for *B. bacteriovorus* species, nor *Bdellovibrio* genus, which instead requires an intra-periplasmic replication for its survival. Only a single study reported a similar epibiotic predation for a different *Bdellovibrio* strain, JSS, which is able to predate only the Gram-negative *Caulobacter crescentus* (Chanyi et al., 2013; Koval et al., 2013; Pasternak et al., 2014), and only another study used Gram-positive species as decoys for *B. bacteriovorus* HD100 in a triple-planktonic predation system (Hobley et al., 2006). Unlike our results (Figure 3), a recent study reported how supernatant of a host-independent (HI) mutant of *B. bacteriovorus* HD100 is able to reduce *S. aureus* biofilm by 75%, but predator itself is not able to do that (Monnappa et al., 2014). Monnappa and colleagues had this evidence on three different *S. aureus* strains, but no direct attack of *B. bacteriovorus* on *S. aureus* was visible. In our study, through FE-SEM, we had the first evidence of predation and survival of strain HD100 preying exclusively a Gram-positive species, with an attack-phase and an epibiotic predation (Figures 2, 4), leading to a diminution of *S. aureus* biofilm of 74% (Figure 3). Morphological changes in the predator/prey “region of contact” are currently under investigation, by means of transmission electron microscopy (TEM) and scanning-transmission electron microscopy (STEM) in our lab. Once depicted by TEM and STEM the intimate contact among *B. bacteriovorus* and *S. aureus*, the forthcoming step will be to study the underlying molecular mechanisms of *S. aureus* killing: due to the short mean predator/prey contact of 185 s, it will be interesting to test if cell wall hydrolysis or protein synthesis interference, as previously observed for *E. coli* (Varon et al., 1969), could be responsible. One could also consider that *B. bacteriovorus* predation on *S. aureus* would be prey strain-dependent, or it would be different if an axenic HI *B. bacteriovorus* is used (Monnappa et al., 2014). In our study we decided to see the actual predatory behavior of *B. bacteriovorus* strain HD100 against *S. aureus*, thus, an axenic HI variant was not used. We thus evidenced a specific interaction of HD100 with *S. aureus* cellular surface (Figure 4C, white arrows), in which the destruction of *S. aureus* cell seemed to occur through the rupture of its membrane and exudation of the cell content (Figure 4C). We also found that *B. bacteriovorus* HD100 was always attached epibiotically to *S. aureus* from the initial to the latter phase of prey destruction. It is conceivable that strain HD100 could use a specific repertoire of bacteriolytic enzymes to rupture *S. aureus* membrane to start exudation. Taking into account the *B. bacteriovorus* HD100 annotated genome (Rendulic et al., 2004), it is noteworthy how this strain owns a huge repertoire of hydrolytic enzymes, such as proteases, glycanases, and DNases. Interestingly, *S. aureus* zymogram showed three lytic bands, at 22, 44 and 67 kDa, among which the last two may represent an additional bacteriolytic activity compared to *P. aeruginosa* zymogram. Further studies are required to understand the mechanism of interaction among *B. bacteriovorus* and Gram-positive species, such as *S. aureus*, along with characterization of lytic enzymes involved and their mode of delivery.

Due to the spread of resistance among clinical pathogens, it was suggested how *B. bacteriovorus* could be used as a “living-antibiotic” (Rendulic et al., 2004; Sockett and Lambert, 2004). It is noteworthy that the dual foraging strategy exhibited by *B. bacteriovorus* on *S. aureus* (epibiotic) and *P. aeruginosa* (periplasmic), would be a favorable condition to actually significantly reduce bacterial loads and established biofilms both in mono- and bi-colonized CF patients, rather than disperse them. Dispersal of alive pathogen bacterial cells, as seen by using specific *B. bacteriovorus*-derived proteases (Monnappa et al., 2014), could be detrimental within an inflamed lung, leaving planktonic cells free to colonize other niches, thus exacerbating CF symptoms. *B. bacteriovorus* was found to be unable to infect mammalian cells (Sockett and Lambert, 2004), and was found in the healthy human gut (Iebba et al., 2013), making it a good candidate to treat infections *in vivo*, and results obtained in this study point toward its use in the treatment of CF pulmonary infections.

## Author contributions

Valerio Iebba, Floriana Santangelo, Antonella Gagliardi, and Luana Ciotoli conceived and performed the experiments, Alessandra Virga, Cecilia Ambrosi, and Monica Pompili performed the zymographic experiments, Valentina Totino, Fabrizio Pantanella and Francesco Mura performed the FESEM imaging and analyses, Laura Selan and Marco Artini performed BioFlux experiments, Riccardo V. De Biase, Maria Trancassini, and Serena Quattrucci provided bacterial strains, Valerio Iebba, Claudio Passariello, Mauro Nicoletti, Lucia Nencioni., and Serena Schippa. wrote the main manuscript text and Valerio Iebba prepared the figures.

### Conflict of interest statement

The authors declare that the research was conducted in the absence of any commercial or financial relationships that could be construed as a potential conflict of interest.
